# Paeoniflorin protects spiral ganglion neurons from cisplatin‐induced ototoxicity: Possible relation to PINK1/BAD pathway

**DOI:** 10.1111/jcmm.14379

**Published:** 2019-06-17

**Authors:** Xiaoyu Yu, Rongjun Man, Yanan Li, Qianqian Yang, Hongrui Li, Huiming Yang, Xiaohui Bai, Haiyan Yin, Jianfeng Li, Haibo Wang

**Affiliations:** ^1^ Shandong Provincial ENT Hospital affiliated to Shandong University Jinan China; ^2^ Department of Otolaryngology Head and Neck Surgery Zibo Central Hospital Zibo China; ^3^ Department of Histology and Embryology, College of basic Medicine Jining Medical University Jinan China

**Keywords:** BAD, paeoniflorin, PINK1, ROS, spiral ganglion neuron

## Abstract

The objective of this study was to elucidate whether paeoniflorin (PF) exerted an effect on cisplatin‐induced spiral ganglion neuron (SGN) damage, with special attention given to the role of PINK1/BAD pathway in this process. Middle cochlear turn culture and C57BL/6 mice were utilized to identify the character of PF in vitro and in vivo. We found that cisplatin treatment led to SGN damage, in which reactive oxygen species (ROS) generation increased, PINK1 expression decreased, BAD accumulation on mitochondria raised and mitochondrial apoptotic pathway activated. Conversely, we demonstrated that PF pre‐treatment obviously mitigated cisplatin‐induced SGN damage. Mechanistic studies showed that PF could reduce ROS levels, increase PINK1 expression, decrease the BAD accumulation on mitochondria and, thus, alleviate the activated mitochondrial apoptosis in SGNs caused by cisplatin. Overall, the findings from this work reveal the important role of PF and provide another strategy against cisplatin‐induced ototoxicity.

## 1 INTRODUCTION

Paeoniflorin (PF), a monoterpene glycoside, is one of the principal bioactive components purified from the dried roots of *Paeonia lactiflora*, which has been widely used in China for about 1200 years*.* 1 Mounting evidence suggests that PF possesses various pharmacological effects such as anti‐depression, anti‐inflammation and neuroprotection.^2^, ^3^, ^4^ PF has also been reported to alleviate oxidative stress,^5^, ^6^, ^7^, ^8^ which can decrease the malondialdehyde (MDA) and lactate dehydrogenase (LDH) leakage, and reduce the expression of NADPH oxidase to decrease reactive oxygen species (ROS) level.^7^, ^9^, ^10^ Moreover, PF also exerts an effect on anti‐apoptosis, which regulates the Bcl‐2 family to reduce cell apoptosis caused by different stimuli, including chemotherapeutic agents.^11^, ^12^, ^13^, ^14^

Cisplatin, one of the most widely used antitumour drugs in clinic,^15^ is greatly limited to clinical application attributable to its serious side effects like ototoxicity and nephrotoxicity. With regard to ototoxicity induced by cisplatin, audiometric studies show that after cisplatin therapy 93% of patients will have a certain degree of progressive and irreversible sensorineural hearing loss.^16^ Previous researches have demonstrated the damage to three major targets, including the stria vascularis, the outer hair cells (HCs) of the organ of Corti and spiral ganglion neurons (SGNs), is the main reason for cisplatin‐elicited hearing loss.^17^ SGNs are specialized bipolar neurons, which transmit the sound input from HCs to the cochlear nucleus and are crucial to hearing. Of note, unlike other ototoxic drugs, the cytotoxic effects of cisplatin in both the organ of Corti and the SGNs are suggested to occur independently of each other as demonstrated by animal models.^17^, ^18^ ROS overproduction, apoptosis and mitochondrial membrane potential alteration are considered to be engaged in cisplatin‐elicited SGN loss,^19^ the precise mechanisms responsible for cisplatin‐elicited SGN damage, however, have not been fully deciphered as yet.

PTEN‐induced putative kinase protein 1 (PINK1) is a serine/threonine‐protein kinase and is necessary for mitochondrial health. It has been well documented that PINK1 can ameliorate the oxidative stress and mitochondrial damage in multiple cells.^20^, ^21^ Recently, we have demonstrated that PINK1 widely exists in SGNs and possesses the protective effect in cisplatin‐induced SGNs injury through inhibiting apoptosis, which implies that PINK1 plays an important role on cisplatin‐induced SGN damage.^22^ Moreover, in other neuronal cells, PINK1 has been reported to regulate the apoptosis‐related gene to increase cell survival.^23^ Bcl‐2 associated death promoter (BAD) is a BH3‐only protein promoting apoptosis through forming heterodimers with the Bcl‐2 and Bcl‐X_L_,^24^ and the physiological activity of BAD is mainly mediated by its phosphorylation. In other neurons, PINK1 has been reported to regulate the BAD phosphorylation to reduce BAD localization on mitochondria, which potentially prevents the apoptotic complex formation and promotes cell survival because increased accumulation of BAD on mitochondria aggravates the apoptosis.^25^, ^26^

To date, the mechanisms of cisplatin ototoxicity have been extensively explored and the anti‐apoptotic and antioxidative effects of PF in different cell types have appeared in literature. However, there is still no report about the role of PF as well as PINK1/BAD pathway on cisplatin‐induced SGN injury. Therefore, this study was designed to elucidate whether paeoniflorin (PF) exerted an effect on cisplatin‐induced SGN damage, with special attention given to the role of PINK1/BAD pathway in this process.

## 2 MATERIALS AND METHODS

### 2.1 Animals and treatments

We purchased the C57BL/6 mice from the Animal Center of Shandong University (Jinan, China) and conducted all animal experiments according to protocols approved by the Animal Care Committee of Shandong University following the guidance for the care and use of Laboratory Animal for Research Purposes. Experiments in vivo studies were implemented on age and sex‐matched postnatal day (P) 30 C57BL/6 mice. 3 mg/kg cisplatin for consecutive 7 days has been widely used in setting up animal models with cisplatin‐induced ototoxicity.^27^, ^28^ It has been reported that intraperitoneal injection with paeoniflorin (15, 30 mg/kg) could suppress cell apoptosis and the anti‐apoptotic effect of PF was better in 30 mg/kg than that in 15 mg/kg.^29^ Therefore, in this work, P30 mice in the control group received normal saline and mice in PF group were given 30 mg/kg PF ip for consecutive 7 days. Mice in cisplatin group received cisplatin injection (3 mg/kg ip) daily for 7 days and mice in PF plus cisplatin group were administered 30 mg/kg PF ip for 2 hours before the daily injection of cisplatin.

### 2.2 Organotypic culture of neonatal mouse cochleae and drug treatment

The C57BL/6 mice were decapitated at P3 and collected only the middle cochlear turn to keep the consistency. Dissection steps and cochlear middle turn culture protocols were conducted as mentioned in previous studies.^27^, ^30^, ^31^ Briefly, we removed the cochlear capsules from the temporal bones to expose the membranous labyrinth, wiped out the stria vascularis and placed the middle turn cochlear explants containing the SGNs onto 10 mm coverslips. According to the previous studies from our laboratory, 50 μmol/L cisplatin treatment for 48 hours induced the most degenerative changes in SGNs compared to the 25 and 150 μmol/L.^27^ Another study has observed that PF at 3, 10 or 30 μmol/L in the media significantly suppresses cell apoptosis and concentrations exceeding 30 μmol/L have no further cytoprotective effect.^32^ Therefore, in our experiments, the middle turn explants in control group received no treatment and explants in PF group were given 30 μmol/L PF for 50 hours. We treated the explants in cisplatin group with 50 μmol/L cisplatin for 48 hours (Sigma). For PF plus cisplatin group, we pre‐treated the explants with 30 μmol/L PF for 2 hours 33 followed by PF plus cisplatin for 48 hours. In vitro study, the proteins for Western blot were extracted from the middle turns, from which the hair cells parts were removed as much as possible.

### 2.3 Tissue preparation for frozen sections

The harvested cochleae were fixed at 4°C overnight with 4% paraformaldehyde and decalcified in 10% EDTA. Then we incubated these samples in 10%, 20% and 30% sucrose and finally the OCT compound (Tissue‐Tek, Sakura Finetek). Subsequently, we adjusted these samples into right position, frozen them on dry ice and cut them into 7 μm sections.

### 2.4 Protein extraction and Western blot analysis

The tissues were collected quickly and lysed with RIPA (Protein Biotechnology, China), which contained the protease inhibitor cocktail (Sigma). Then, we used the BCA Protein Assay Kit (Protein Biotechnology) to estimate the protein concentration. Lysates were separated on 10% SDS‐PAGE gels and transferred to PVDF membrane, which were blocked with milk and incubated with primary antibodies β‐actin (1:2000, ZSGB‐BIO, China), Bax (1:1000, Cell Signaling Technology), cleaved caspase‐3 (1:1000, Cell Signaling Technology), Bad (1:1000, Abcam) and PINK1 (1:1000, Abcam) overnight at 4°C. The immunoblots were detected by the ECL kit (Santa Cruz) and Image Laboratory analysis software (Bio‐Rad).

### 2.5 Immunofluorescence staining

The procedures were performed as previously mentioned^33^ and the samples were incubated with the primary antibodies mentioned blow: anti‐NeuN (1:1000, Cell Signaling Technology), anti‐Bad (1:500, Abcam) and anti‐Tuj 1 (1:1000, Neuromics). Then, on the following day, we treated the samples with secondary antibody (1:1000, Invitrogen) along with DAPI (1:800, Invitrogen).

To detect mitochondrial ROS levels, we used Mito‐SOX Red staining (Life Technologies) on the basis of the manufacturer's instructions. And we took advantage of the TUNEL assay (Invitrogen) to detect apoptosis.

### 2.6 Spiral ganglion neuron counting

We used Image J software to quantify the immunostaining positive cells. In vivo study, we measured the relative length of the scale bar (20 μm) in the figures and set scale to 20 μm in the Image J software. Then, we calculated the triangular area where SGN localized and used the ‘Point selections’ tool in the Image J to count the immunostaining positive cells. The SGN density per unit area = total immunostaining positive cell number/total triangular area of SGN. In vitro study, SGNs in which the nucleus comprised 40% of the soma area were counted in each optical section using the Image J software. The total number of SGNs in each spiral ganglion explant was obtained by summing the SGN counts in all consecutive sections, and the SGN density per unit area was calculated as mentioned before.

### 2.7 Statistical analyses

The data we shown were the summary from at least three individual experiments and were presented as the mean ± SD. We performed a one‐way ANOVA followed by a LSD's multiple comparison test when comparing more than two groups. And statistical significance was determined at *P* < 0.05.

## 3 RESULTS

### 3.1 PF ameliorates cisplatin‐elicited SGN loss in vivo

**Figure 1:**
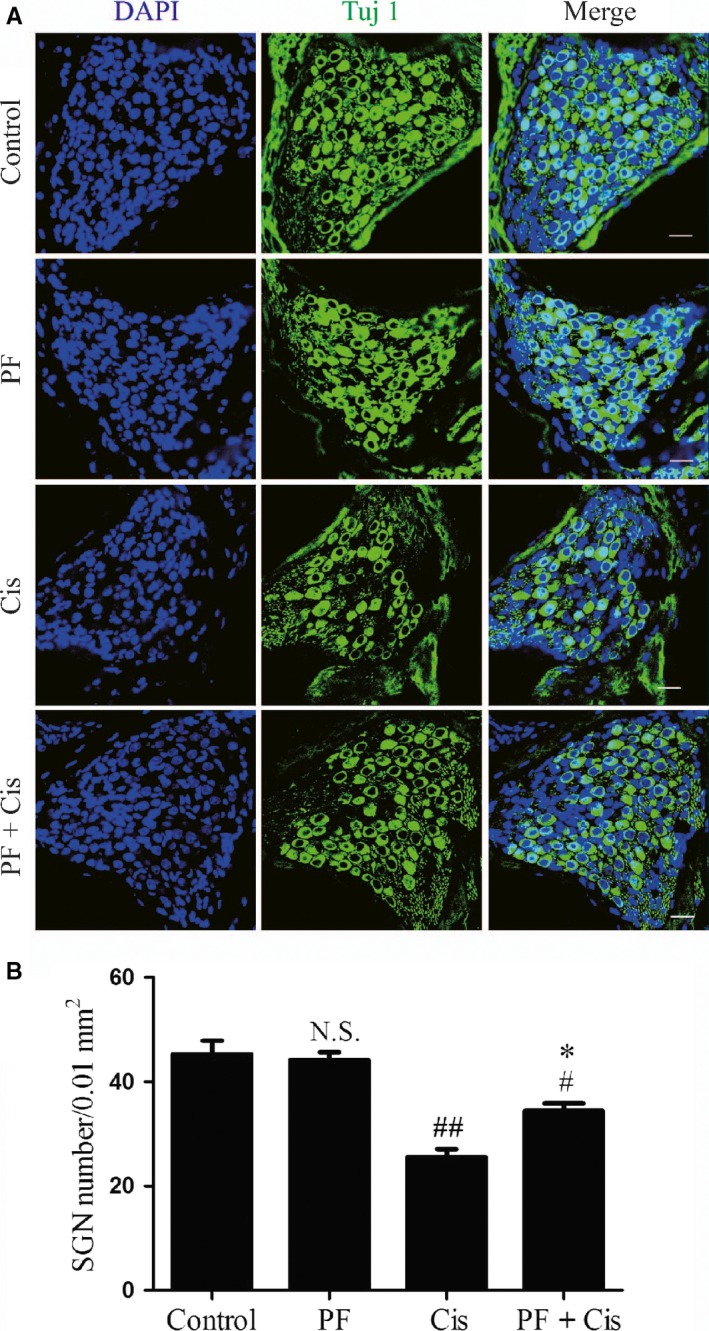
PF ameliorates cisplatin‐elicited SGN loss in vivo. A, DAPI (blue) and Tuj 1 (green) double staining in the middle‐turn cochlear sections of control, PF, cisplatin and PF plus cisplatin group. B, Tuj 1‐positive cell quantification in the middle‐turn cochlear sections from each group. #*P* < 0.05, ##*P* < 0.01 vs control group, *P < 0.05 vs cisplatin group, n = 3. Scale bars = 20 μm

After treatment for 7 consecutive days, the ABR thresholds in cisplatin group were significantly increased than those in control group as shown in Figure S1. And then the cochleae were dissected out and the middle‐turn cochlear sections were selected as the representative. We used Tuj 1 as the SGNs marker and found that there was no significant difference between the control group and the PF group with regard to the morphology and survival number of SGNs. Immunofluorescence results showed that there was obvious SGN loss in the cisplatin group in contrast to the high‐density SGNs in the control group, while SGNs with PF pre‐treatment exhibited less loss than those in cisplatin group (Figure 1A). Then we calculated the Tuj 1‐positive cells in middle‐turn cochlear sections and detected that in cisplatin group the mean density of SGNs was decreased contrasted to the control group (##*P* < 0.01, n = 3). In PF pre‐treated mice, the SGN survival was decreased compared to that in the control group but increased than the group submitted to cisplatin only (#*P* < 0.05, *P < 0.05, n = 3) (Figure 1B).

### 3.2 PF alleviates the cisplatin‐elicited SGN injury in vitro

**Figure 2:**
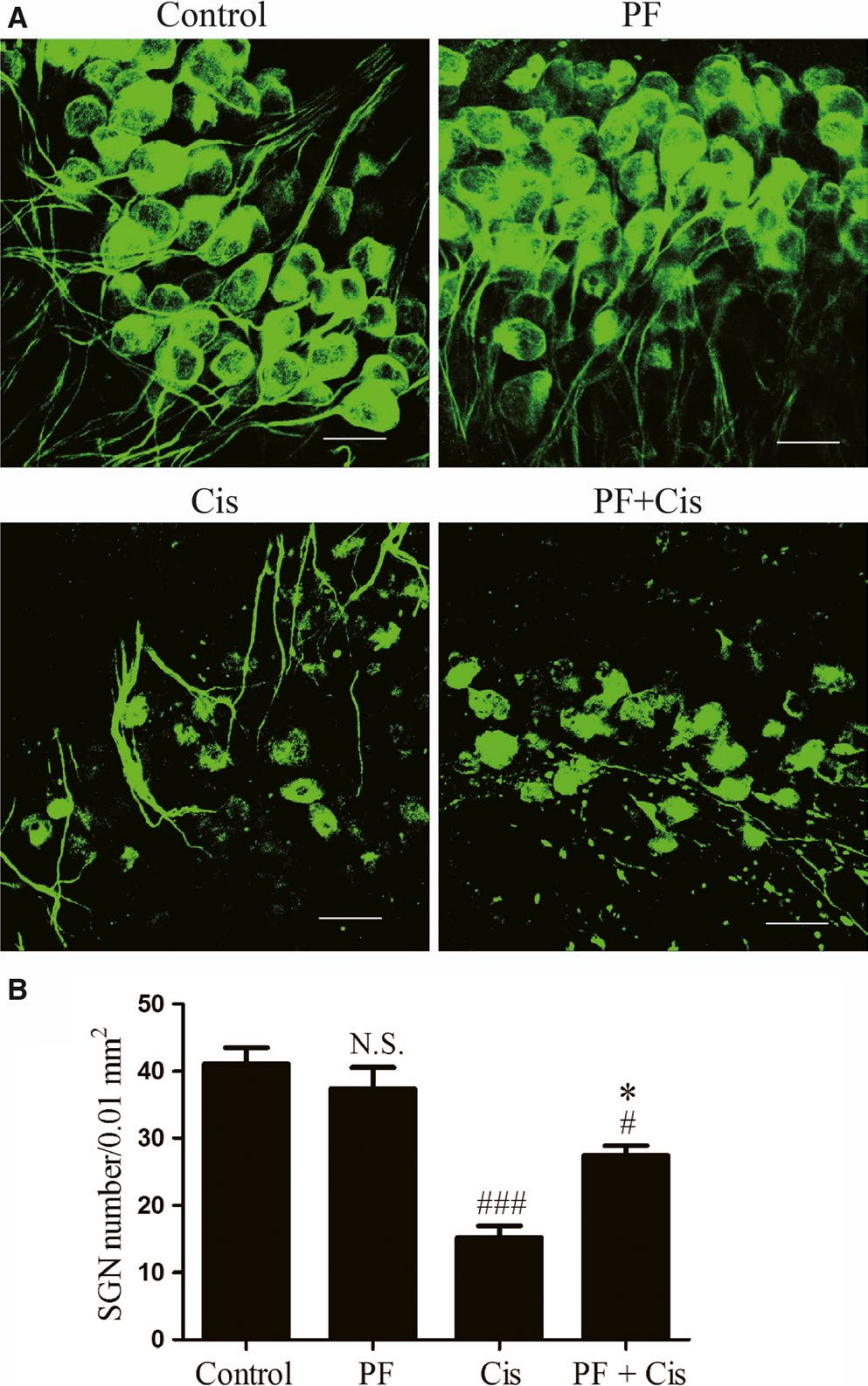
PF alleviates the cisplatin‐elicited SGN injury in vitro. A, Immunostaining of Tuj 1 (green) in the middle‐turn cochlear explants of control, PF, cisplatin and PF plus cisplatin group. B, Tuj 1‐positive cell quantification in the middle turn explants of each group. #*P* < 0.05, ###*P* < 0.001 vs control group, *P < 0.05 vs cisplatin group. n = 4. Scale bars = 20 μm

According to the previous study, we treated the middle turn explants with the 50 μmol/L cisplatin for 48 hours. The somata of SGNs in control group and PF group were large and round or oval‐shaped with intense Tuj 1 cytoplasm labelling and weak nucleus labelling. The peripheral auditory nerve fibres (ANFs), which protrude out radially from SGNs to HCs, were also labelled with Tuj 1 and organized into smooth and thick fascicles. In the cisplatin group, the number of SGNs were obviously decreased, the somata of the remaining SGNs were shrunken and shrivelled and the ANFs were fragmented and in disordered arrangement. However, in PF plus cisplatin group, the SGNs showed less loss and the somata were larger compared to the group only received cisplatin (Figure 2A). Subsequently, we counted the SGNs survival and found that the SGN survival in cisplatin group was remarkably reduced (###*P* < 0.001, n = 4) while the PF pre‐treated group showed less SGN loss contrasted to the group only received cisplatin (*P < 0.05, n = 4) (Figure 2 B).

### 3.3 PF extenuates the cisplatin‐induced SGN apoptosis in vivo

**Figure 3:**
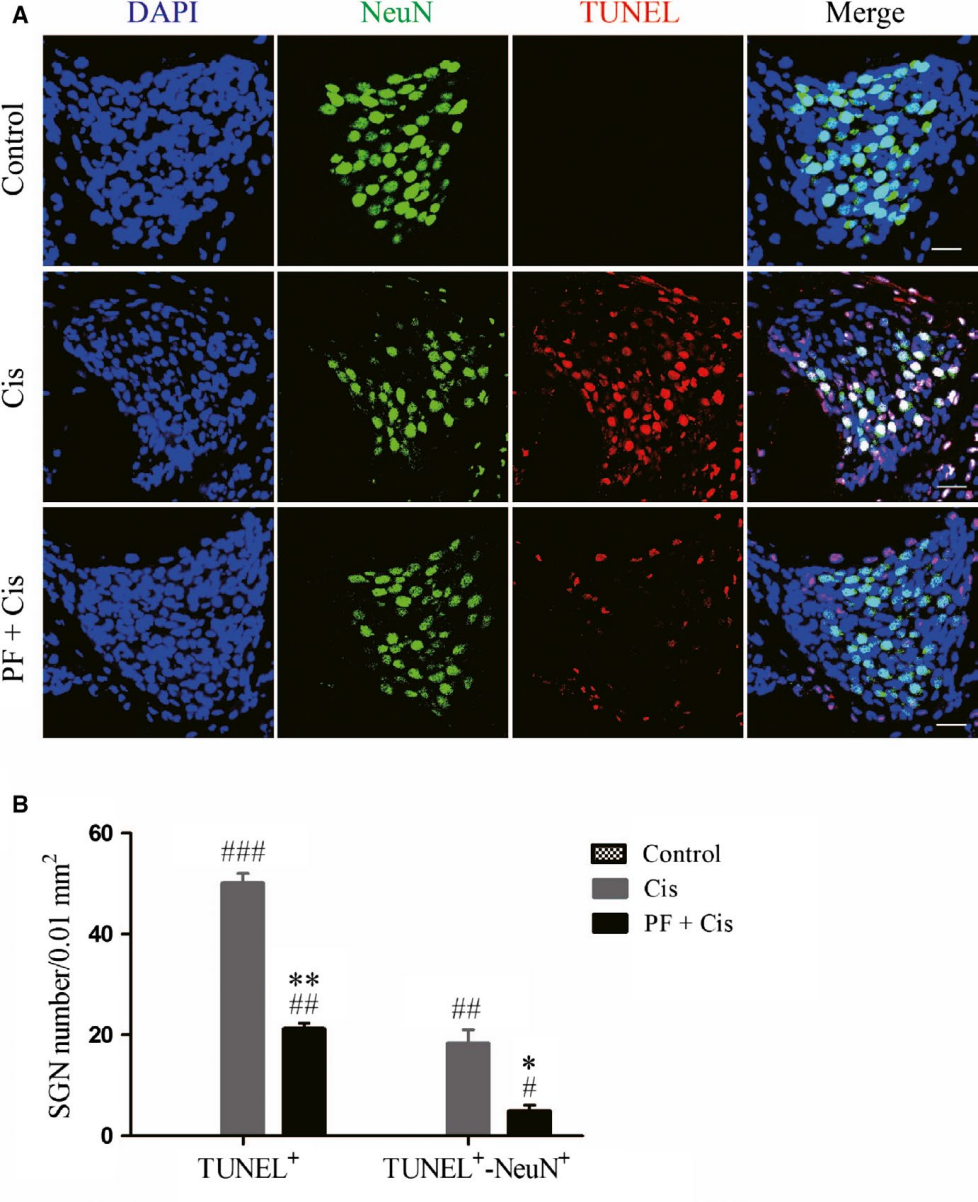
PF extenuates the cisplatin‐induced SGN apoptosis in vivo. A, TUNEL staining (red) in the middle‐turn cochlear sections from control, cisplatin and PF plus cisplatin group. NeuN (green) was applied as SGN marker. B, TUNEL‐positive and TUNEL/NeuN double‐positive cell quantification in each group. #*P* < 0.05, ##*P* < 0.01, ###*P* < 0.001 vs control group, *P < 0.05, **P < 0.01 vs cisplatin group. n = 4. Scale bars = 20 μm

We used TUNEL assay to detect SGN apoptosis in middle‐turn cochlear sections and used NeuN to label SGNs. In cisplatin group, strongly TUNEL positive staining could be found in SGNs and supporting cells, while PF plus cisplatin group showed less positive staining (Figure 3A). The TUNEL/NeuN double‐positive and TUNEL‐positive cells were dramatically enhanced in explants subjected to cisplatin only (##*P* < 0.01, ###*P* < 0.001, n = 4), whereas these numbers in PF pre‐treated mice were remarkably reduced contrasted to the group only received cisplatin (*P < 0.05, **P < 0.01, n = 4) (Figure 3B).

### 3.4 PF protects SGNs against the cisplatin‐induced apoptosis in vitro

**Figure 4:**
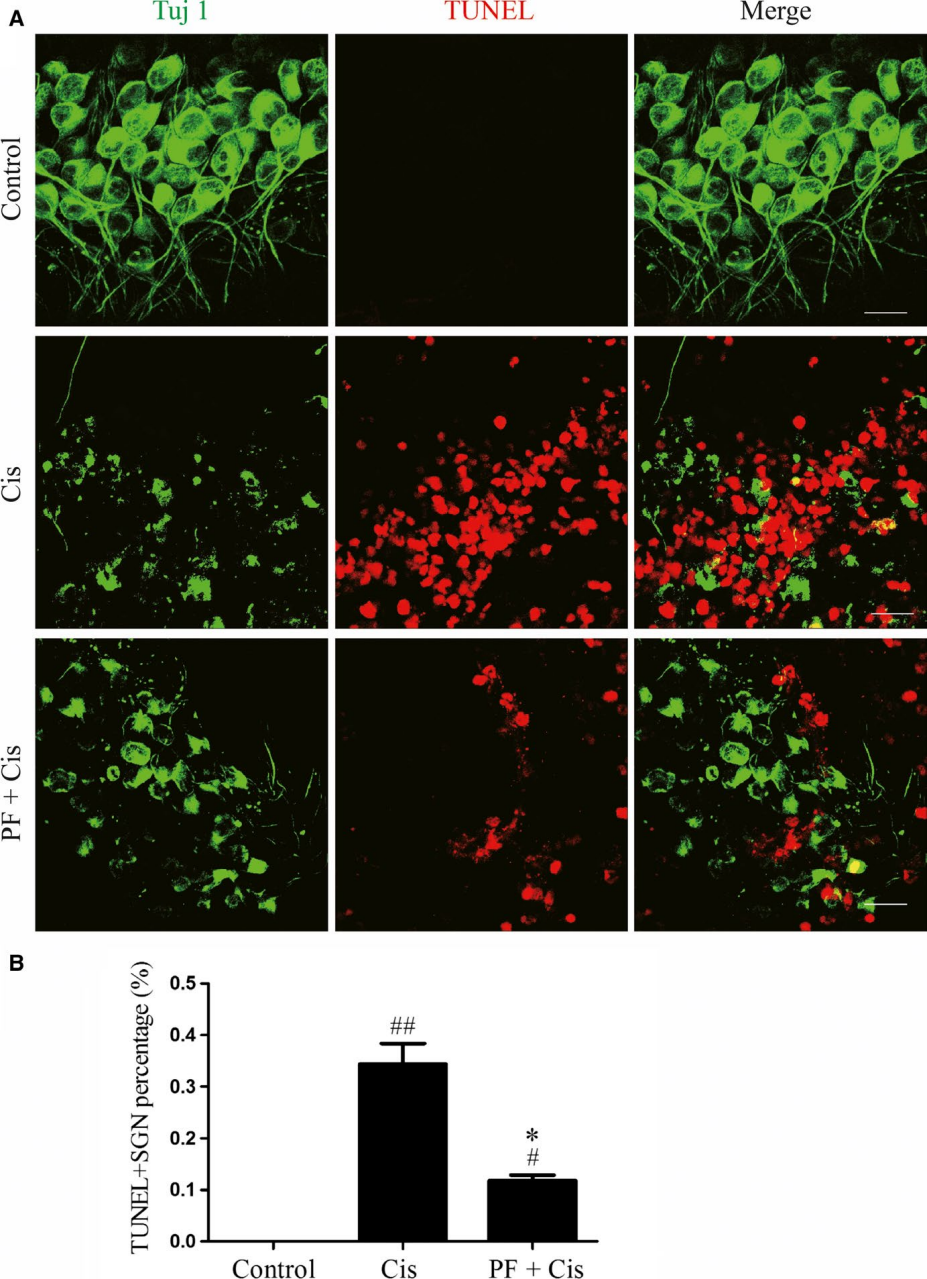
PF protects SGNs against the cisplatin‐induced apoptosis in vitro. A, TUNEL staining (red) in the middle turn explants of the control, cisplatin and PF plus cisplatin groups. Tuj 1 (green) was applied as the SGN marker. B, The TUNEL‐positive SGN percentages (the number of TUNEL/Tuj 1 double positive SGNs divided by the total Tuj 1 cells) in each group. #*P* < 0.05, ##*P* < 0.01 vs control group, *P < 0.05 vs cisplatin group, n = 4

We used TUNEL assay to measure the cisplatin‐induced apoptosis in SGNs. SGNs that were double labelled by Tuj 1 and TUNEL were considered to be apoptotic SGNs and there was no double‐positive SGN in control group. We found the TUNEL/Tuj 1 double‐positive cell percentage was raised in the explants only received cisplatin (##*P* < 0.01, n = 4) while this percentage was reduced contrasted to the group subjected to cisplatin only (*P < 0.05, n = 4) (Figure 4A,B).

### 3.5 PF inhibits the mitochondrial apoptotic pathway both in vivo and in vitro

**Figure 5:**
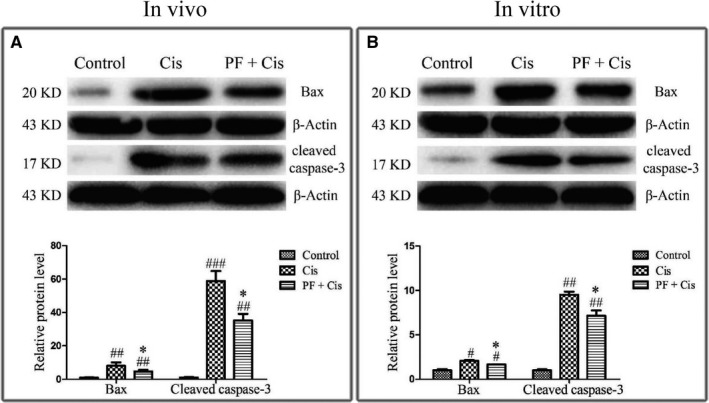
PF inhibits the mitochondrial apoptotic pathway both in vivo and in vitro. A, In vivo study, the protein levels of Bax and cleaved caspase‐3 in the control, cisplatin and PF plus cisplatin groups. B, In vitro study the protein levels of Bax and cleaved caspase‐3 in the control, cisplatin and PF plus cisplatin group. β‐Actin served as the loading control in each lane. # *P* < 0.05, ## *P* < 0.01, ### *P* < 0.001 vs control group, *P < 0.05 vs cisplatin group. n = 3

Given that the aforementioned results suggesting apoptosis is activated in cisplatin‐induced SGN damage as well as PF can ameliorate cisplatin‐elicited apoptosis, we subsequently explored the mechanism underlying the protective effect of PF in cisplatin ototoxicity. Western blot showed that in cisplatin group Bax and cleaved caspase‐3 were remarkably raised in vivo and in vitro (#*P* < 0.05, ##*P* < 0.01, ###*P* < 0.001, n = 3), whereas in PF pre‐treatment group Bax and cleaved caspase‐3 were reduced contrasted to the group only subjected to cisplatin (*P < 0.05) (Figure 5A,B)*.*

### 3.6 PF mitigates the BAD activation in SGNs after cisplatin exposure

**Figure 6:**
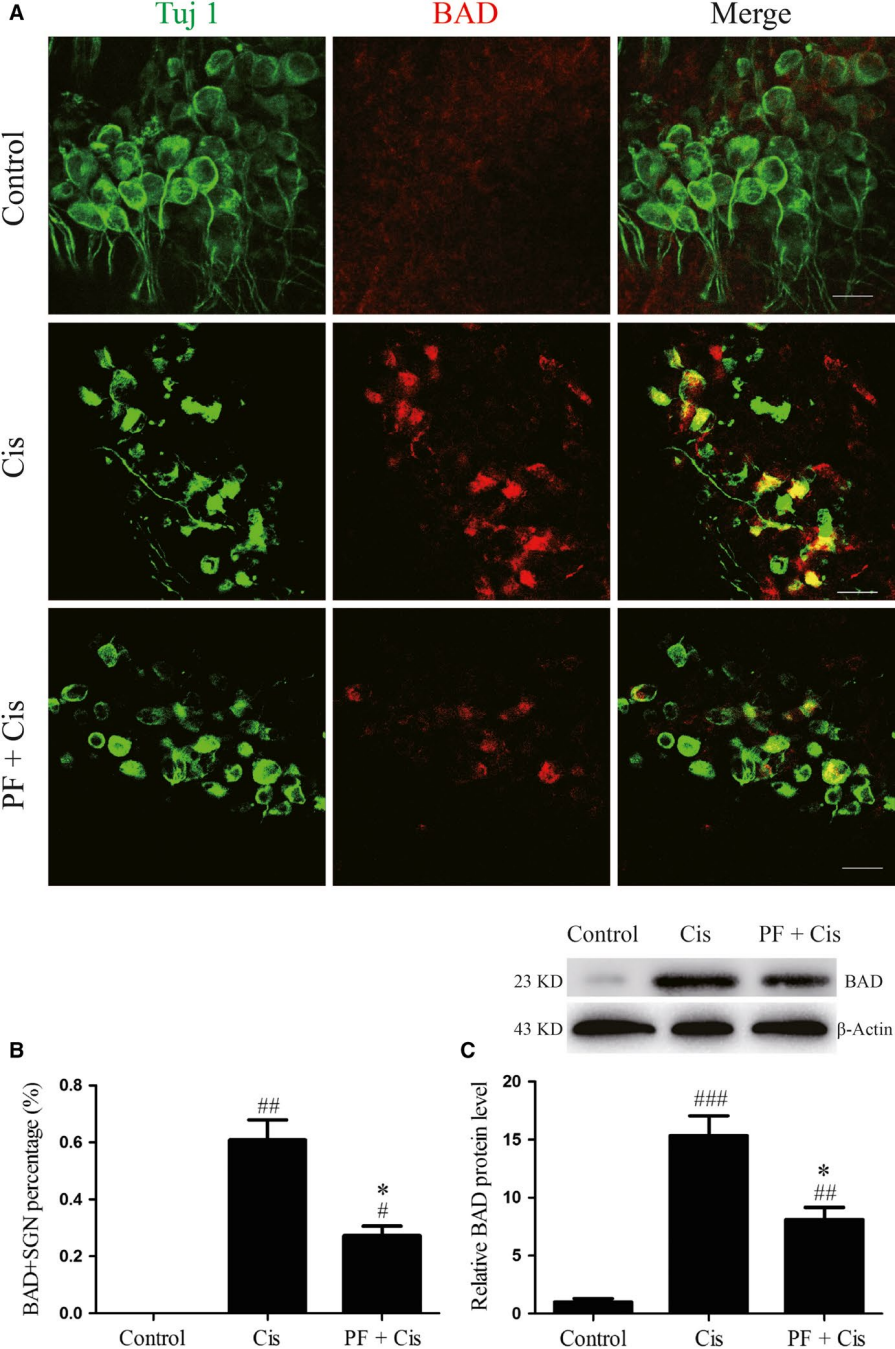
PF mitigates the BAD activation in SGNs after cisplatin exposure. A, Tuj 1 (green) and BAD (red) double staining in the middle turn cochlear explants of control, cisplatin and PF plus cisplatin groups. Tuj 1 (green) was applied as the SGN marker. Scale bars = 20 μm. B, The BAD‐positive SGN percentages (the number of BAD/Tuj 1 double positive SGNs divided by the total Tuj 1 cells) in each group. C, The relative protein level of BAD in each group. β‐Actin served as the loading control in each lane. #*P* < 0.05, ##*P* < 0.01, ###*P* < 0.001 vs control group, *P < 0.05 vs cisplatin group. n = 4

Next, we investigated the expression of BAD through immunofluorescence and Western blot. Immunofluorescence results showed that in control group the fluorescence of BAD was very weak, while in cisplatin group the expression of BAD was strongly positive and distributed in a spotty manner around the nuclei, where typically mitochondria are clustered in the cell. In PF plus cisplatin group, the immunostaining of BAD was relatively weaker than that in cisplatin group (Figure 6A). We also found that in cisplatin group the BAD/Tuj 1 double‐positive cell percentage was remarkably raised (##*P* < 0.01, n = 4) while in PF pre‐treatment group the percentage was reduced than that in the cisplatin group (*P < 0.05, n = 4) (Figure 6A,B). Moreover, BAD protein level was dramatically enhanced in cisplatin group contrasted to the control group (###*P* < 0.001, n = 4), while the BAD expression in PF plus cisplatin group was reduced than the group subjected to cisplatin only (*P < 0.05, n = 4) (Figure 6C).

### 3.7 PINK1 is relatively increased in PF pre‐treated SGNs after cisplatin insult

**Figure 7:**
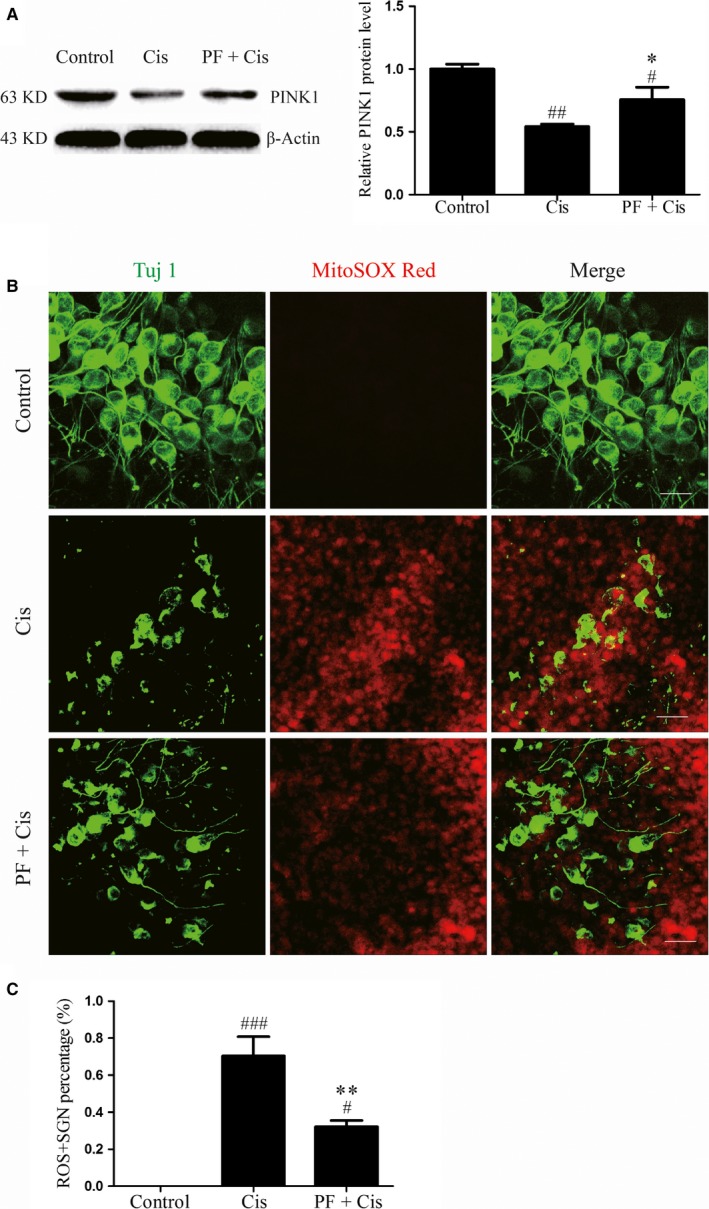
PTEN‐induced putative kinase protein 1 (PINK1) expression is increased and mitochondrial ROS levels are reduced in PF plus cisplatin group. A, The PINK1 expression was reduced in cisplatin group but increased in PF plus cisplatin group as demonstrated by Western blot. β‐Actin served as the loading control in each lane. n = 4. B, Mito‐SOX (red) and Tuj 1 (green) double staining in the control, cisplatin and PF plus cisplatin group. Tuj 1 (green) was used as the SGN marker. Scale bars = 20 μm. B, The Mito‐SOX Red‐positive SGN percentages (the number of Mito‐SOX Red/Tuj 1 double positive SGNs divided by the total Tuj 1 cells) in each group. #*P* < 0.05, ##*P* < 0.01, ###*P* < 0.001 vs control group. *P < 0.05, **P < 0.01 vs cisplatin group*.* n = 4

Analysis of the protein level of PINK1 in different groups showed that the PINK1 expression was remarkably decreased in cisplatin group (##*P* < 0.01, n = 4), while the PINK1 expression was increased in PF plus cisplatin in comparison to the group subjected to cisplatin only (*P < 0.05, n = 4) (Figure 7A).

### 3.8 PF pre‐treatment reduces mitochondrial ROS levels in SGNs after cisplatin insult

Lastly, Mito‐SOX red staining was applied to estimate the ROS level of SGNs, and we found that in cisplatin group the immunofluorescence was more apparent in contrast to the PF pre‐treatment group. We also calculated the Mito‐SOX Red‐positive SGN percentage in each group and found that in cisplatin group the percentage was increased (###*P* < 0.001, n = 4) (Figure 7C), whereas this percentage was decreased in PF pre‐treatment group than the group subjected to cisplatin only (**P < 0.01, n = 4) (Figure 7C).

## 4 DISCUSSION

In the present study, we found that SGNs in control and PF group had no significant difference in terms of morphology and survival number. This indicates that PF itself at concentration of 30 mg/kg, ip or 30 μmol/L has no cytotoxicity to SGNs. SGNs exposed to cisplatin exhibited extensive degeneration, shrunken somata as well as fragmented and disordered arrangement of ANFs as demonstrated by immunofluorescence, and cisplatin treatment significantly decreased the SGN survival in vivo and in vitro, both of which verify the cytotoxic effects of cisplatin on SGNs, which are in accordance with our recent findings.^27^, ^28^ Conversely, PF pre‐treatment preserved the greater morphology of SGNs and increased the SGN survival in comparison with the cisplatin group, indicating that PF can preserve the SGNs against cisplatin‐induced damage.

As the SGN apoptosis is the main mechanism responsible to cisplatin‐induced SGN damage,^27^, ^28^, ^34^ we subsequently used TUNEL assay to examine the role of PF on apoptosis. And we found that in cisplatin group, the percentages of TUNEL‐positive and TUNEL/Tuj 1 or NeuN double‐positive cells were significantly raised, which suggests that cisplatin induced SGN death mainly via apoptosis. PF also exerts an effect on anti‐apoptosis in different works.^11^, ^12^ In this study, we found less TUNEL positive staining in PF pre‐treated group than the group only received cisplatin, implying that PF protects SGNs from cisplatin via inhibiting apoptosis*.*

Subsequently, we attempted to determine the mechanisms underlying the protection provided by PF on cisplatin‐induced SGN apoptosis. Available data have demonstrated that cisplatin induced the auditory cell death through activation of mitochondrial apoptosis pathway.^28^, ^35^, ^36^ The pro‐apoptotic protein Bax changes the permeability of mitochondrial membranes, causes the cytochrome c release and then activates the downstream caspase‐3 by forming cleaved caspase‐3, which triggers mitochondrial apoptosis. We found that Bax and cleaved caspase‐3 were increased in cisplatin group, implying that cisplatin induces SGN death mainly through mitochondrial apoptotic pathway. However, in PF plus cisplatin group, the Bax and cleaved caspase‐3 were obviously decreased, indicating that PF can inhibit the mitochondrial apoptotic pathway to mitigate the cisplatin‐induced SGN apoptosis.

BAD, the BH3‐only protein, belongs to the pro‐apoptotic Bcl‐2 family and its phosphorylation takes an important effect in mitochondrial apoptosis.^37^ In the current study, immunostaining and Western blot results showed that after cisplatin treatment BAD was obviously increased and distributed in a spotty manner around the nuclei, where typically mitochondria are clustered in the cell. These results indicate that after cisplatin insult BAD is activated and increases mitochondrial location in SGNs, potentially allowing the formation of apoptotic complex, activating the caspase‐3 and promoting mitochondrial apoptosis. It has been confirmed that PF could inhibit the activation of BAD in other tissues.^11^, ^12^ In this work, the BAD expression in PF pre‐treated group was reduced in contrast to cisplatin group, implying that PF can restrain the BAD pathway to alleviate the activated mitochondrial apoptosis in SGNs after cisplatin exposure.

It has been well‐established that PINK1 can regulate the phosphorylation of mitochondrion‐localized BAD, thus preventing cell apoptosis,^25^ and PINK1 possesses the protective effect in cisplatin‐induced SGNs injury.^22^ In our study, PINK1 expression in cisplatin group was reduced contrasted to the control group, and we speculate that as the PINK1 expression decreases, there is corresponding down‐regulation of BAD phosphorylation and increased accumulation of BAD on mitochondria, thereby resulting in activation of mitochondrial apoptosis in SGNs. In PF plus cisplatin group, the protein level of PINK1 was relatively increased, suggesting that PF can interfere with the PINK1/BAD pathway to protect SGNs against cisplatin.

As the cisplatin‐induced SGN apoptosis is closely linked with the ROS accumulation and PINK1 can be affected by ROS,^22^ we speculate that ROS overproduction contributes to decreased PINK1 expression in cisplatin‐induced SGN damage and PF can alleviate oxidative stress in this process to protect SGNs. In this regard, we evaluated the ROS levels in SGNs after cisplatin intervention and found the increased ROS levels in SGNs exposed to cisplatin, suggesting that cisplatin treatment can aggravate oxidative stress in SGNs, possibly resulting in decreased PINK1 expression. However, the PF plus cisplatin group showed reduced ROS levels than the cisplatin group, which indicates that PF can ameliorate the oxidative stress in SGNs after cisplatin.

Also, we have made a statistical analysis between the control and the PF plus cisplatin group and found that there are still significant differences between them in terms of SGN survival, SGN apoptosis and so on. These results indicate that PF can exert partial protection against cisplatin‐induced SGN damage but cannot protect the SGNs completely from the cisplatin. Although ROS overproduction and apoptosis are widely considered to be responsible for the cisplatin‐induced ototoxicity, there are still many other pathways involved, such as mitochondrial membrane potential alteration, Wnt pathway and autophagy.^19^ Moreover, all the mechanisms underpinning cisplatin‐induced ototoxicity have not been fully explained yet, which need to be fully studied further.

In conclusion, we have demonstrated that cisplatin‐elicited SGN damage is closely linked with ROS overproduction and PINK1 down‐regulation, which, in turn, increase the accumulation of BAD on mitochondria, activate the caspase‐3 and promote mitochondrial apoptosis. Interestingly, PF can reduce ROS levels, increase the PINK1 expression and reduce the BAD accumulation on mitochondria, and thus preserve SGNs from cisplatin damage. Overall, the findings from this work reveal the important role of PF and provide another strategy against cisplatin‐induced ototoxicity.

## CONFLICT OF INTEREST

The authors confirm that there is no conflict of interests.

## Data Availability

The data that support the findings of this study are available from the corresponding author upon reasonable request.
