# Supplementary material for: Paeoniflorin protects spiral ganglion neurons from cisplatin‐induced ototoxicity: Possible relation to PINK1/BAD pathway
Source: J Cell Mol Med. 2019 Jun 17;23(8):5098–107. doi: 10.1111/jcmm.14379 (PMC6653418; doi:10.1111/jcmm.14379)
Supplement: Supplementary file 1 [file JCMM-23-5098-s001.docx]

Paeoniflorin protects spiral ganglion neurons from cisplatin-induced ototoxicity: possible relation to PINK1/BAD pathway


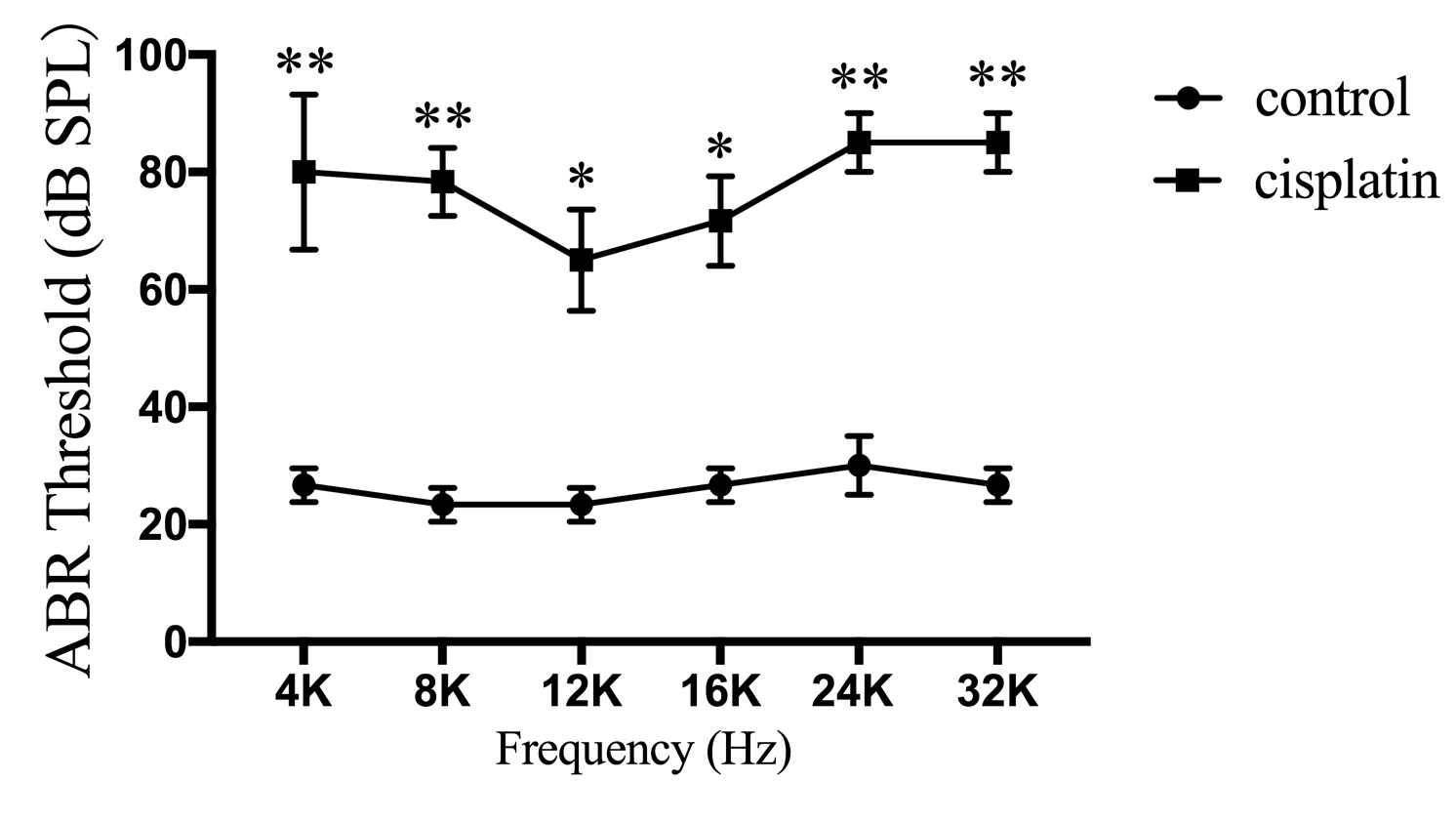


Supplemental Fig. 1. The ABR thresholds in control group and cisplatin group. * *p* < 0.05, ** *p* < 0.01 *versus* control group. n = 4.
